# Combinatorial Treatment with Praziquantel and Curcumin Reduces *Clonorchis sinensis* Parasite Burden and Clonorchiasis-Associated Pathologies in Rats

**DOI:** 10.3390/pharmaceutics16121550

**Published:** 2024-12-03

**Authors:** Soon-Ok Lee, Ki Back Chu, Keon-Woong Yoon, Su In Heo, Jin-Ho Song, Jianhua Li, Sung-Jong Hong, Fu-Shi Quan

**Affiliations:** 1 Department of Medical Zoology, School of Medicine, Kyung Hee University, Seoul 02447, Republic of Korea; amkey68@khu.ac.kr; 2 Medical Research Center for Bioreaction to Reactive Oxygen Species and Biomedical Science Institute, School of Medicine, Graduate School, Kyung Hee University, Seoul 02447, Republic of Korea; 3 Department of Parasitology, College of Medicine, Inje University, Busan 47392, Republic of Korea; kbchu@inje.ac.kr; 4 Department of Infectious Disease and Malaria, Paik Institute of Clinical Research, Inje University, Busan 47392, Republic of Korea; 5 Department of Biomedical Science, Graduate School, Kyung Hee University, Seoul 02447, Republic of Korea; ky20200310318@khu.ac.kr (K.-W.Y.); hsi7200@khu.ac.kr (S.I.H.); 6 Department Pharmacology, College of Medicine, Chung-Ang University, Seoul 06974, Republic of Korea; jinhos@cau.ac.kr; 7 State Key Laboratory for Diagnosis and Treatment of Severe Zoonotic Infectious Diseases, Key Laboratory for Zoonosis Research of the Ministry of Education, Institute of Zoonosis, College of Veterinary Medicine, Jilin University, Changchun 130062, China; 8 Chung-Ang University College of Medicine, Seoul 06974, Republic of Korea; hongsj@cau.ac.kr

**Keywords:** *Clonorchis sinensis*, curcumin, fibrosis, mitochondrial activity, praziquantel

## Abstract

**Background/Objectives**: Clonorchiasis is a foodborne parasitic disease that can lead to severe biliary fibrosis and cholangiocarcinoma. While praziquantel (PZQ) is available for clonorchiasis treatment, it cannot revert the histopathological damage incurred through parasite-induced fibrosis. Curcumin (CUR) is an emerging experimental drug possessing anti-inflammatory and fibrosis-alleviating effects, thus signifying its potential as an anthelmintic drug. Here, we evaluated the effect of CUR+PZQ combinatorial drug treatment on *C. sinensis* infection as well as its effect on ameliorating fibrotic tissue damage in rats. **Methods**: Worm viabilities following CUR and PZQ treatments were confirmed through microscopy and tetrazolium salt absorption. Anthelminthic effect and hepatobiliary damage mitigation in rats were determined by quantifying worm recovery, histopathological staining, and enzyme-linked immunosorbent assay. **Results**: CUR+PZQ at LD_50_ doses demonstrated a time- and dose-dependent antiparasitic effect in vitro, which was markedly greater than either drug alone. Rats were infected with *C. sinensis*, and drugs were administered at 1 and 4 weeks post-infection (wpi) to assess drug-induced changes in worm burden. Significant reductions in worm burden recoveries were observed following CUR+PZQ treatment at both time points, accompanied by markedly reduced serum and mucosal IgG responses. ALT and AST levels were also substantially lower in combinatorial drug treatment groups than controls. Histopathological examinations confirmed that parasite-induced bile duct lumen widening and liver fibrosis were suppressed at 1 wpi, implying that CUR+PZQ co-treatment can alleviate clonorchiasis-associated pathologies. **Conclusions**: Our findings indicate that CUR+PZQ co-treatment improved parasite clearance and promoted the resolution of hepatobiliary tissue damage resulting from chronic clonorchiasis.

## 1. Introduction

Clonorchiasis is an infectious disease caused by the trematode *Clonorchis sinensis*, which can thrive in the bile ducts of hosts for more than 20 years. *C. sinensis* infection results in chronic bile duct damage via several mechanisms, including mechanical stress, exposure to *C. sinensis* excretory-secretory antigens, the host’s inflammatory immune response, and others. This consequently leads to cholangitis, formation of biliary calculus, hepatobiliary fibrosis, and even cholangiocarcinoma if left untreated [1,2]. In 2009, the International Agency for Research on Cancer categorized this pathogen as a group I carcinogen [3]. Praziquantel (PZQ) is currently the drug of choice for treating clonorchiasis and other liver fluke infections. However, the short-term adverse events associated with PZQ, such as inflammatory cell influx and enhanced oxidative stress that contribute to bile duct fibrosis, remain unresolved [4,5]. Furthermore, PZQ is a racemic mixture comprising enantiomers where only one form exhibits an antiparasitic effect that is limited to the adult stages of trematodes [6]. For this reason, a novel therapeutic regimen that can effectively eliminate parasites while reducing undesirable adverse effects such as fibrotic damage is urgently needed. One such strategy to address the aforementioned problem is co-administration of PZQ with an antioxidant.

Studies attempting to identify novel drugs capable of reverting the hepatobiliary sequelae with enhanced anthelmintic efficacy are ongoing. One such candidate is curcumin (CUR), an antioxidant derived from the natural compound turmeric that has demonstrated its anthelmintic effect against *Schistosoma mansoni* [7,8]. CUR was also reported to inhibit hepatic fibrosis, which is thought to involve an apoptotic mechanism and suppression of hepatic stellate cells [9]. Similarly, CUR treatment reduced periductal fibrosis development in hamsters infected with the liver fluke *Opisthorchis* spp. [10,11]. Currently, several studies have reported that combinatorial treatment involving PZQ and antioxidants has been efficacious against several trematodes. Treating *S. mansoni*-infected mice with pumpkin seed oil co-formulated with PZQ not only reduced the parasite burden but fibrotic tissue damage as well [12]. Nanoencapsulated CUR and PZQ co-treatment also reduced the periductal fibrosis caused by *O. viverrini* infection [13]. However, most of the CUR and PZQ co-administration studies reported to date targeted the genera *Schistosoma* or *Opisthorchis*, while their effects on *C. sinensis* infection remain largely unexplored.

To address this loophole in our understanding, we recently investigated the potential of CUR as an alternative treatment option for clonorchiasis [14]. Our findings revealed that CUR exerted an anthelmintic effect against both the adult and larval stages of *C. sinensis*, while PZQ was mostly effective against the adult stage. Furthermore, PZQ treatment could not revert the fibrotic damage caused by *C. sinensis* infection, whereas collagen deposition occurred to a lesser extent upon CUR treatment. Based on these findings, we reasoned that supplementing PZQ with CUR would not only enhance trematicidal efficacy but also ameliorate *C. sinensis*-induced hepatobiliary complications. Here, we investigated the anthelmintic effect of CUR and PZQ co-treatment and explored whether this approach could mitigate the fibrotic damage caused by *C. sinensis* infection in rats. Findings from our investigation unveiled an alternative strategy that could potentially be applied in clinical settings to suppress hepatobiliary fibrosis during clonorchiasis treatment.

## 2. Materials and Methods

### 2.1. Chemical Reagents

Commercially available CUR and PZQ were purchased from Cayman Chemical (Ann Arbor, MI, USA) and Sigma-Aldrich (P4668; St. Louis, MO, USA), respectively. Concentrated stock solutions of CUR and PZQ were prepared following the manufacturer’s guidelines by dissolving the powders in either deionized water or dimethyl sulfoxide (DMSO). Solutions were aliquoted and stored at −20 °C until use. Fluorescein diacetate (FDA) and propidium iodide (PI) were purchased from Sigma-Aldrich to calculate worm viability. Liver alanine aminotransferase (ALT) and aminotransferase (AST) quantities were measured using enzyme-linked immunosorbent assay (ELISA) kits (ab285264, ab263883; Abcam, Cambridge, UK). Tetrazolium salt (XTT), phenazine methosulfate (PMS), and M199 media without phenol red pH indicator were purchased from Sigma-Aldrich.

### 2.2. C. sinensis Metacercaria and Juvenile Worm Collection

*C. sinensis* metacercaria (CsMC) and newly excysted juvenile worms (CsNEJs) were collected from infected *Pseudorasbora parva*. After digesting *P. parva* in a 1% pepsin–HCl solution at 37 °C for 3 h, the contents were centrifuged, and the pelleted portions were carefully washed with saline solution. The CsMC were carefully isolated under the microscope, resuspended in Locke’s solution, and stored at 4 °C until use. Fresh CsNEJs were prepared by treating the CsMC with 0.01% trypsin (BD Biosciences, Franklin Lakes, NJ, USA).

### 2.3. Anthelmintic Effect of CUR and PZQ Treatment Against CsMC and CsNEJs

In a 96-well plate, 50 CsMC or CsNEJs were added to the respective wells. CUR, PZQ, and CUR+PZQ at concentrations of 12.5, 25, 50, and 100 μM were inoculated into the wells as described in our previous study [14]. A 1% DMSO solution was used as the vehicle control. The survival of the CsMC and CsNEJs was monitored at 24 h intervals for 3 days using a Leica DMi8 microscope (Leica, Wetzlar, Germany). The LC_50_ of CUR and PZQ against both life cycle stages of *C. sinensis* was determined, and these values were used to find the optimal concentration for CUR+PZQ combinatorial treatment [14]. Cell viabilities were calculated as described elsewhere using the following equation [15]:

$$\mathrm{Viability}\left(\%\right)=\frac{\mathrm{Live}\;\mathrm{worms}\;(\mathrm{FDA})}{\left[\mathrm{Live}\;\mathrm{worms}\left(\mathrm{FDA}\right)+\mathrm{Dead}\;\mathrm{worms}\left(\mathrm{PI}\right)\right]}\times 100$$

### 2.4. Viability Measurement of Adult Parasites Using Tetrazolium Salt (XTT) Absorption

Adult parasite viabilities were calculated following incubation with 100 or 250 μM of CUR, PZQ, or CUR+PZQ for 72 h using the XTT-based method described elsewhere [16]. Briefly, XTT powder at a concentration of 1 mg/mL was dissolved in the M199 medium at 55 °C. To enhance XTT reduction, PMS was included, which was prepared by dissolving in phosphate-buffered saline (PBS) at 0.383 mg/mL. Afterward, XTT and PMS were combined at a 50:1 ratio, and parasites were seeded at seeding densities of 25, 50, 75, and 100 per well. Absorbance readings at 450 nm were measured using a SpectraMax M5 microplate reader (Molecular Devices, San Jose, CA, USA), and regression lines were drawn. Adult worm viabilities were calculated using the equation derived from the regression lines.

### 2.5. Evaluating the Combinatorial Treatment Efficacy of CUR+PZQ In Vivo

All experimental procedures were approved by Kyung Hee University IACUC (permit ID: KHUASP-24-052). Female Sprague-Dawley rats aged 5 weeks were purchased from Central Lab. Animal Inc. (Seoul, Republic of Korea). Upon arrival, rats were randomly grouped (n = 4 per group) and housed in an approved animal facility with a 12 h day and night cycle. Rats had easy, free access to food and water. To evaluate the anthelmintic effects of the drugs on both juvenile and adult worms, rats were orally infected with 50 CsMC, and infected rats were orally treated at either 1 or 4 weeks post-infection (wpi). Animals were grouped as follows: week 1 infection control (G1), week 4 infection control (G2), 250 mg/kg CUR (G3), 250 mg/kg PZQ (G4), 250 mg/kg CUR+PZQ (G5), 250 mg/kg CUR (G6), 250 mg/kg PZQ (G7), 250 mg/kg CUR+PZQ (G8), and naïve (G9). Rats were sacrificed by CO_2_ inhalation at 4 weeks post-treatment in a 10 L CO_2_ chamber. Briefly, a CO_2_ flow rate of 5 L per minute was maintained for 5 min until respiratory cessation and corneal opacification were observed. CO_2_ flow was maintained for another 3 min, and cervical dislocation was performed to ensure rats were properly euthanized. Blood samples of the sacrificed rats were collected by cardiac perforation using a syringe, incubated at RT for 30 min, and centrifuged at 1000× *g*, 4 °C for 10 min. Sera in the supernatant layer were collected and stored at −20 °C until use. Rat liver ALT and AST were measured to assess liver function using ELISA kits as previously described [17]. Results were presented as units per liter (U/L). Morphometric and histopathologic analyses were also performed to evaluate liver fibrosis and its associated pathologies. Briefly, rat livers were carefully excised and bile duct thickness was measured before further processing. *C. sinensis* worms in the bile ducts were collected and enumerated under a microscope. The right lobes of the livers were fixed in 10% formalin for histopathological observation. To assess mucosal antibody response, 10 cm of the duodenum starting directly below the pyloric sphincter was collected from each rat. Tissues were longitudinally cut to expose the intestinal lumen and incubated in 2 mL PBS at 37 °C for 4 h. The contents were centrifuged at 1000× *g* for 30 min for mucosal sample collection. The parasite burden reduction rate was calculated as follows:

% = [infection control mean − drug treatment mean]/infection control mean × 100

### 2.6. Histopathological Staining of Liver Tissues

Rats were sacrificed at 4 and 8 wpi, and the right lobes of the liver tissues were collected for histopathological studies as previously described [14]. In brief, paraffin-embedded liver tissues were deparaffinized and subsequently stained with hematoxylin and eosin. After placing mounting media (Thermo Fisher, Waltham, MA, USA) on the tissue cross-sections, coverslips were overlaid, and images were observed under the microscope. Picrosirius red (PSR) staining was also performed to evaluate liver fibrosis using a Picrosirius red staining kit (Abcam) following the manufacturer’s instructions. Collagen fibers stained in red were calculated under a microscope as described [18].

### 2.7. C. sinensis-Specific Antibody Detection

ELISA was performed using sera and mucosal antibody samples of rats as previously described [14]. *C. sinensis* excretory-secretory proteins (ESPs) were harvested from adult worms cultured in serum-free Dulbecco’s modified Eagle medium, and these were used as coating antigens. Briefly, 96-well plates were coated overnight at 4 °C with 5 μg/mL of ESPs in carbonate coating buffer. The next day, PBS containing 0.1% Tween-20 (PBST) was used to wash the wells. After blocking with 1% bovine serum albumin at 37 °C, rat serum samples (1:100 dilution in PBST) or undiluted mucosal samples were added to the respective wells. After 1 h of incubation at 37 °C, anti-rat IgG-HRP (1:2000 dilution in PBST; Innovative Research, Novi, MI, USA) was added, and the plates were incubated for another 1 h at 37 °C. The o-phenylenediamine substrate was added to each well, and reactions were stopped with diluted sulfuric acid. Absorbance readings at 450 nm were measured using a microplate reader (Molecular Devices).

### 2.8. Statistical Analyses

All animal samples were individually processed, and data are expressed as the mean ± SD. Statistical analyses were performed using Prism 6.0 software (GraphPad, Boston, MA, USA). The means of each group were compared, and statistical significance was determined using either the one-way ANOVA with Bonferroni’s *post hoc* test or Student’s *t*-test. *p* values less than 0.05 were considered statistically significant, and this was denoted using asterisks (* *p* < 0.05, ** *p* < 0.01, *** *p* < 0.001).

## 3. Results

### 3.1. Experimental Scheduling for the Combinatorial Drug Treatment Evaluation

**Figure 1:**
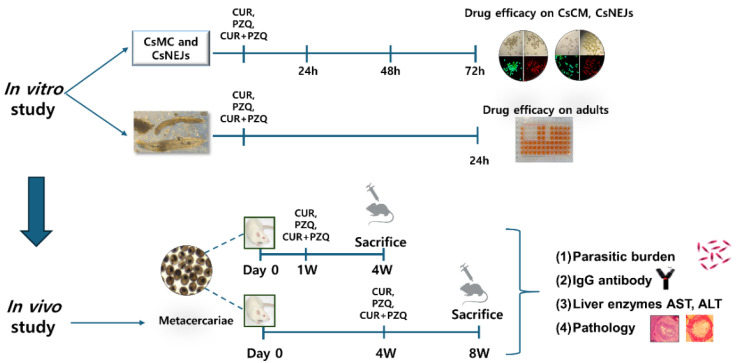
A schematic overview of the experimental scheduling of in vitro and in vivo studies.

An overview of the experiments involved in this study was provided (Figure 1). CUR, PZQ, and CUR+PZQ efficacies against both larval and adult *C. sinensis* were evaluated under in vitro and in vivo conditions. Worm viabilities were assessed through microscopy and XTT methods. For an in vivo evaluation, rats were infected with 50 CsMC and treated at designated time points. Blood and other tissue samples were collected to determine the overall efficacy of the CUR+PZQ regimen. Different rat groups were assessed for liver fibrosis using fibrosis-related parameters, including liver function tests, hepatic histopathology, and morphometric measurements of tissues.

### 3.2. Evaluating the Time- and Dose-Dependent Effects of CUR and PZQ Co-Treatment

**Figure 2:**
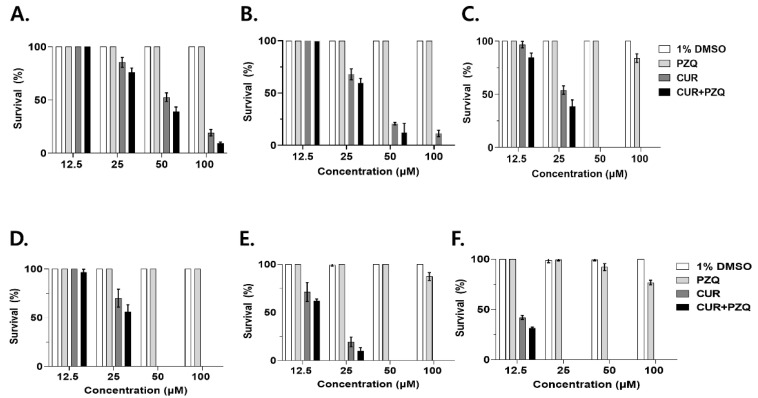
CsMC and CsNEJ survival upon CUR, PZQ, and CUR+PZQ treatment. The CsMC were treated with CUR, PZQ, and CUR+PZQ for 24 h (**A**), 48 h (**B**), and 72 h (**C**). CsNEJs were also treated with identical drugs for 24 h (**D**), 48 h (**E**), and 72 h (**F**). Experiments were performed in triplicate, and representative dose- and time-dependent data are provided.

The CsMC were treated with CUR, PZQ, and CUR+PZQ at various doses for up to 72 h. A dose- and time-dependent relationship between CUR and CsMC survival was observed against CsMC (Figure 2A–C). After 24 h of CUR treatment, survival rates were 100, 85.3, 52.7, and 19.3% for 12.5, 25, 50, and 100 μM, respectively. Prolonged exposure to CUR for 48 and 72 h resulted in a marked reduction in survival. Co-administering CUR and PZQ enhanced the larvicidal effect against CsMC at all doses and time points. CsMC survival resulting from CUR+PZQ treatment was comparable to CUR treatment alone. In contrast, PZQ did not exert any larvicidal effect throughout all the tested time points. Specifically, the survival rate only fell below 100% when the CsMC were treated with 100 μM PZQ for 72 h. An identical approach was used to test the efficacy against CsNEJs using CUR, PZQ, and CUR+PZQ (Figure 2D–F). CUR treatment strongly affected CsNEJs in a dose- and time-dependent manner. At CUR concentrations exceeding 50 μM, none of the drug-treated parasites survived at any time point. PZQ did not demonstrate trematicidal effects during the first 24 h at any concentrations. Survival fell below 100% when the CsNEJs were treated with 100 μM for 48 h and longer or at 50 μM for 72 h. CUR+PZQ treatment against CsNEJs was efficacious, similar to CUR alone. Overall, CUR+PZQ demonstrated greater larvicidal effects than CUR or PZQ alone at all time points, but significant differences between CUR and CUR+PZQ were not detected.

### 3.3. Fluorescent Microscopic Analysis of Anthelmintic Drug Efficacy

**Figure 3:**
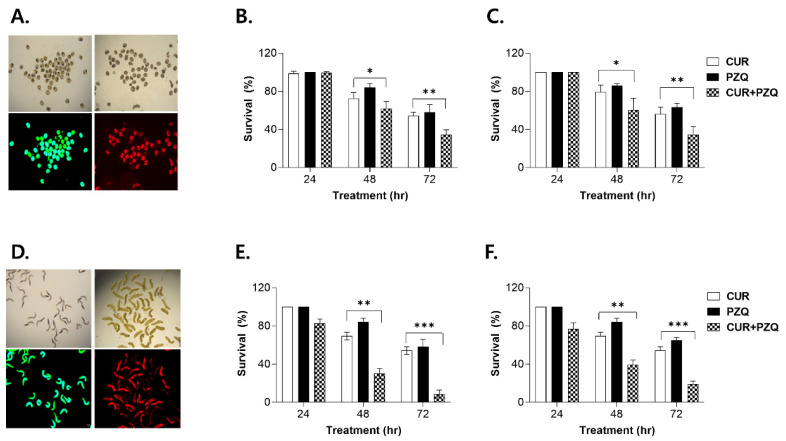
Fluorescent microscopic analysis of CsMC and CsNEJ viability. The CsMC were treated with LC_50_ doses of CUR, PZQ, and CUR+PZQ for 24, 48, and 72 h. Representative images depicting viable (green) and dead (red) CsMC with respective brightfield photographs are provided (**A**). Viable and dead CsMC are enumerated using brightfield microscopy (**B**) and fluorescence microscopy (**C**). Identical approaches were used to enumerate the number of viable CsNEJs. Representative drug-treated CsNEJs are provided (**D**), along with viability counts using light microscopy (**E**) and fluorescence-based methods (**F**). Statistical significance is indicated using asterisks (* *p* < 0.05, ** *p* < 0.01, *** *p* < 0.001). Images for (**A**,**D**) were acquired under 100× magnification.

Prior to the CUR+PZQ combinatorial treatment efficacy evaluation, optimal doses for the two drugs were determined by measuring their LC_50_ values against CsMC and CsNEJs. The LC_50_ doses for CUR and PZQ were determined to be 21.7 μM and 320.4 μM for CsMC and 12.1 μM and 320.4 μM for CsNEJs, respectively. Representative images for drug-treated CsMC were provided (Figure 3A). CsMC viabilities were observed using brightfield and fluorescent microscopy. Larval motility within the cyst wall, as well as distinct suckers and excretory bladders, were observed in viable CsMC. However, in dead CsMC, contours for the suckers were difficult to distinguish, and excretory bladders were less intact. Larval motility was also absent in dead CsMC. These viable and dead CsMC were fluorescently labeled and visualized as green and red, respectively. The survival data acquired using both brightfield microscopy and fluorescence-based techniques were similar (Figure 3B,C). At 24 h of treatment, CsMC survival was 100% for all treatment groups. However, with prolonged exposure time, survival gradually declined. Significant differences in survival became noticeable, with survival in the 72 h treatment group being markedly lower than its respective counterpart at 48 h. Notably, differences between CUR and CUR+PZQ and between PZQ and CUR+PZQ were observed after 48 h. Similar to the CsMC, discernible morphological differences were also observed between live and dead CsNEJs, such as the absence of a dark-stained excretory bladder (Figure 3D). Survival trends for the drug-treated CsNEJs were similar to those observed in the CsMC with minor differences (Figure 3E,F). While neither CUR nor PZQ elicited a larvicidal effect at 24 h, CUR+PZQ co-treatment led to a partial reduction in survival. By 48 h post-treatment, a significant reduction in survival was observed for CUR+PZQ, and this trend continued to 72 h as well.

### 3.4. XTT-Based Adult Worm Viability Calculation

**Figure 4:**
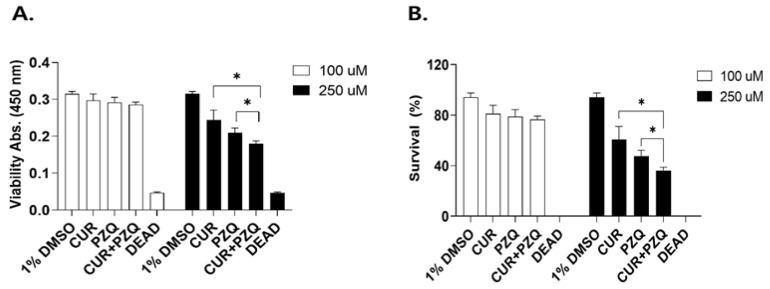
XTT-based viability evaluation of *C. sinensis*. Adult *C. sinensis* worm viabilities were determined using XTT. Parasite viabilities and survival under two different drug doses were determined after 72 h of treatment (**A**,**B**). Statistical significances are indicated using asterisks (* *p* < 0.05).

Next, we incubated *C. sinensis* adult worms with either 100 or 250 μM of CUR, PZQ, and CUR+PZQ for 72 h and measured the resulting absorbances at 450 nm. At 100 μM, changes in the three drug treatment groups were negligible compared to the 1% DMSO vehicle control (Figure 4A). However, noticeable differences were observed when the worms were treated with 250 μM. Compared to the 100 μM groups, increasing the drug concentration to 250 μM induced a significantly greater anthelmintic effect that reduced the overall survival for all three treatment groups. Among the experimental groups, the lowest absorbance readings were detected from parasites incubated with 250 μM of CUR+PZQ. Compared to the survival data at 100 μM treatment, the mean survival rates for CUR, PZQ, and CUR+PZQ were significantly reduced when parasites were treated with 250 μM (Figure 4B). Of note, combining CUR+PZQ led to significantly greater worm viability reduction than PZQ alone. These findings indicate that combinatorial CUR+PZQ is an effective anthelmintic, and its efficacy can be validated using the XTT method.

### 3.5. Worm Burden, IgG Antibody Responses, and ALT and AST Responses

**Figure 5:**
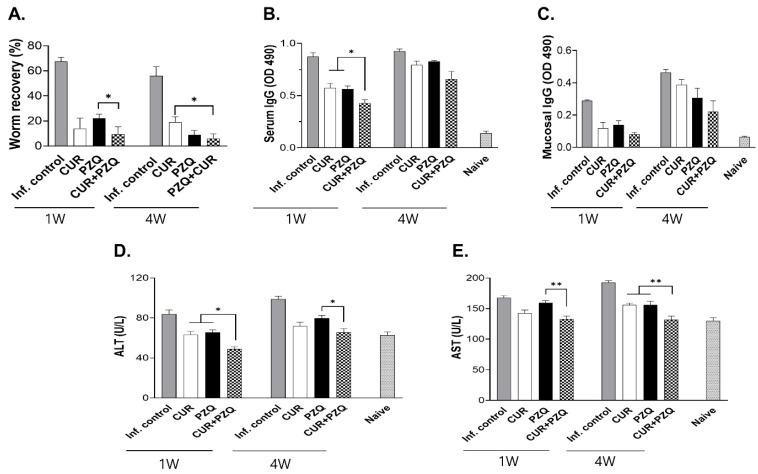
Worm burden, antibodies, and liver damage assessment. Samples were harvested following two different treatment time points. Adult *C. sinensis* worms were recovered from the bile ducts of rats and quantified to calculate worm recovery (**A**). ELISA was performed using the sera (**B**) and duodenal samples (**C**) of rats to determine *C. sinensis*-specific antibody responses. ALT (**D**) and AST (**E**) concentrations in serum samples were evaluated. Statistical significances are indicated using asterisks (* *p* < 0.05, ** *p* < 0.01).

Infected rats were treated with CUR, PZQ, or CUR+PZQ at 1 or 4 wpi and sacrificed after 1 month. The parasite burden in the bile ducts of the rats was quantified (Figure 5A). The worm recovery in the infection control was 67.5%, but drug treatment at 1 wpi significantly lessened the worm burden. The worm recovery rates for CUR, PZQ, and CUR+PZQ were 14%, 22%, and 9.5%, respectively. Significant differences were also observed between PZQ and CUR+PZQ. Upon treatment at 4 wpi, the worm recovery rates were 18.5%, 9%, and 6% for CUR, PZQ, and CUR+PZQ, respectively. As with the 1 wpi treatment, all three treatments resulted in a significantly lower worm recovery rate than the infection control, with CUR+PZQ being significantly more effective than CUR alone. CUR+PZQ led to significantly less worm recovery than PZQ at 1 wpi, but not at 4 wpi. Serum antibody responses against *C. sinensis* antigens were also evaluated (Figure 5B). At 1 week post-treatment, antibody responses were significantly lower than those of the infection control. The parasite-specific antibody response induced by CUR+PZQ was significantly less than either CUR or PZQ at 1 wpi. However, in rats that received treatment at 4 wpi, the antibody responses were comparable. In contrast to serum IgG, significant differences were not observed at either time point (Figure 5C). While CUR+PZQ elicited the lowest antibody induction, the means were not significantly different compared to the other treatment groups. To test liver functionality, the ALT and AST levels of rats were evaluated. *C. sinensis* infection led to enhanced ALT levels, but drug treatment restored the ALT concentration to near basal levels irrespective of the treatment time point (Figure 5D). Significant differences between the treatment groups were detected, especially between PZQ and CUR+PZQ at both time points. AST responses were significantly heightened following *C. sinensis* infection, but drug treatment reduced these to a small extent (Figure 5E). Similar to the ALT levels, combining CUR+PZQ was associated with significant reductions in AST compared to the PZQ control.

### 3.6. Gross and Histopathological Examination of Rat Liver

**Figure 6:**
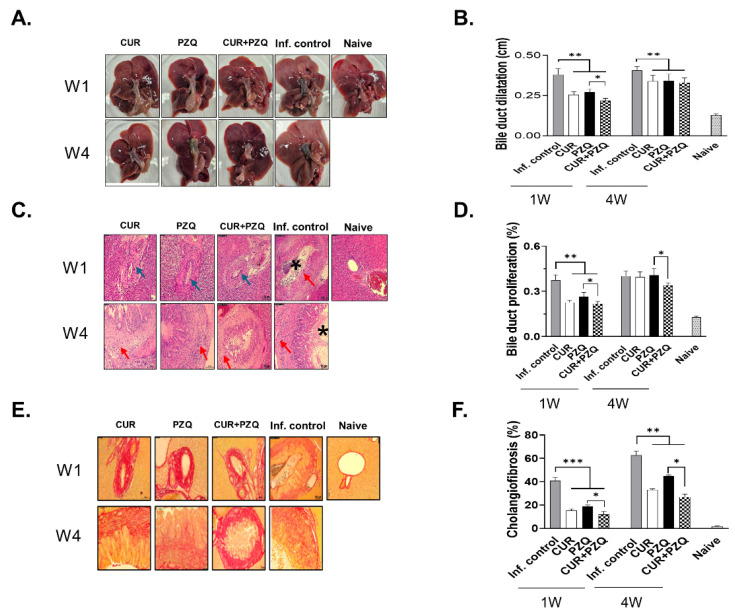
Liver histopathology assessment. Liver samples were harvested and used to perform histopathological analyses. Representative liver tissue images from each group (**A**), along with the bile duct dilations of rats, are provided (**B**). H&E staining showing the presence of *C. sinensis* worms and inflammatory cellular influx and parasites (**C**). Bile duct cellular hyperplasia due to chronic clonorchiasis was measured (**D**). Representative PSR staining images and cholangiofibrosis under different drug treatment conditions (**E**,**F**). Statistical significances are indicated using asterisks (* *p* < 0.05, ** *p* < 0.01, *** *p* < 0.001). Asterisk, *C. sinensis* adult; blue arrow, inflammatory cellular influx; red arrow, fibrosis. All images were acquired under 200× magnification.

The liver tissues of sacrificed rats were carefully excised, and pathological features were observed (Figure 6A). As shown in the representative images, distinct abnormalities in the liver tissues were not observed across all groups. However, the bile ducts of the infection controls were relatively thicker than those of the drug treatment groups and possessed a significantly larger number of adult worms. To confirm the presence of fibrosis, bile duct dilations were measured (Figure 6B). Bile ducts of the infection controls were characterized by inflammatory cellular influx, substantial fibrosis, and widened lumen. However, their severity was significantly reduced in rats that received the drug treatment at 1 wpi. CUR+PZQ administration resulted in significantly lesser bile duct dilation than PZQ. At 4 wpi, drug treatment significantly reduced the bile duct dilation but to a lesser extent than observed at 1 wpi due to the presence of adult parasites. To evaluate the pathological damage mitigation conferred by CUR+PZQ, H&E staining was performed (Figure 6C). In the infection control, parasites embedded in the bile ducts were detected at both time points. Extensive inflammatory cellular influx near the bile ducts was detected with fibrosis and hyperplasia. Substantial reductions in hepatic fibrosis were observed in the treatment groups. When drugs were administered at 1 wpi, significantly less bile duct proliferation was observed compared to the infection control (Figure 6D). A significant difference in bile duct hyperplasia was observed between PZQ and CUR+PZQ. However, when treated at 4 wpi, neither CUR nor PZQ had a profound effect on inhibiting bile duct hyperplasia. Only CUR+PZQ treatment lessened this to a significant extent, especially when compared to PZQ. Collagenous contents in the bile ducts were confirmed by PSR staining. Extensive fibrosis of the bile duct and its surroundings was detected in the infection control, which was substantially diminished in the drug treatment groups (Figure 6E). Fibrosis was much more prominent in the infection control at 4 wpi. Substantial cholangiofibrosis was observed, especially in the infection control rats (Figure 6F). When rats were treated at 1 wpi, cholangiofibrosis was significantly reduced. Compared to PZQ, a significantly lesser degree of cholangiofibrosis was observed following CUR+PZQ treatment. This was also the case for rats that received treatments at 4 wpi. CUR+PZQ treatment appeared to be the most effective of the three drug treatments, as indicated by the significantly reduced cholangiofibrosis compared to the PZQ control.

## 4. Discussion

The present study investigated the anthelmintic efficacy of CUR+PZQ combinatorial treatment as well as its potential to limit parasite-induced fibrosis. We found that CUR+PZQ effectively reduced the worm burden and prevented the formation of extensive fibrosis and inflammation in the bile ducts of rats, signifying its developmental potential as an alternative treatment option for clonorchiasis.

Many of the findings presented in this study are consistent with the findings reported elsewhere. Serum ALT and AST are well-known biomarkers of hepatic damage, and evidence of *C. sinensis* enhancing serum ALT and AST has been documented [19,20]. Furthermore, mitigation of hepatic inflammation and liver fibrosis by CUR has been reported [21]. Consistent with these findings, ALT/AST responses were significantly enhanced in the infection control. However, in contrast, ALT and AST levels were retained at near-basal levels in the treatment groups, thus implying that CUR+PZQ lessened the *C. sinensis*-induced hepatobiliary damage. In our previous study, anti-*C. sinensis* serum antibody responses were comparable across all treatment groups, and significant differences were not detected compared to the infection control group [14]. Similar findings were observed in the present study, albeit with minor differences. At 1 wpi, CUR+PZQ treatment led to significantly lower parasite-specific antibody induction than either CUR or PZQ alone. While antibody responses were also lowered at 4 wpi, the differences in antibody responses were negligible. Previously, one earlier study reported that changes in serum antibody levels following PZQ treatment are correlated with the parasite worm burden [22]. Another study reported that serum *C. sinensis*-specific antibody responses were positively correlated with the infection intensity, with sex differences contributing to the immunological milieu [23]. Based on these reports, we reasoned that the lower antibody induction in CUR+PZQ reflected the aforementioned phenomenon, as combinatorial treatment led to a significantly lower worm burden than either CUR or PZQ alone.

Interestingly, drug-induced worm burden reduction did not necessarily correlate with reduced liver pathology in the treatment groups. At 1 wpi, administering drug treatment led to a substantial reduction in worm recovery in CUR+PZQ compared to PZQ. This was accompanied by a significant reduction in bile duct dilation at 1 wpi. However, these changes were not reflected in rats receiving drug treatment at 4 wpi. Despite CUR+PZQ treatment resulting in significantly greater worm burden reduction than CUR or PZQ alone, differences in bile duct dilation or cellular proliferation were comparable for all three treatment groups at 4 wpi. While conflicting findings were observed for bile duct dilation, bile duct hyperplasia, and cholangiofibrosis findings in drug-treated rats, they correlated with the worm burden. Evidently, co-administering CUR along with PZQ lessened the severity of fibrosis in rats at both time points. While deciphering CUR’s anti-fibrotic mechanism of action was beyond the scope of this study, findings reported by several research groups suggest that this process involves the inhibition of hepatic stellate cell activation and several signal cascades [24,25]. Alternatively, though speculative, the antioxidant properties of CUR may have played a role. In support of this notion, combining PZQ with the antioxidant N-acetyl-L-cysteine substantially reduced hepatic granulomatous inflammation induced during experimental schistosomiasis [26]. Furthermore, during malaria, parasite infection induces mitochondrial oxidative stress that ultimately results in hepatocyte apoptosis, and this can be prevented by intervening with antioxidants such as melatonin [27,28]. Unfortunately, the mechanism of action responsible for CUR-mediated inhibition of clonorchiasis remains largely elusive and should be investigated in future studies. In our present study, the PZQ dose (250 mg/kg) was selected based on the findings reported elsewhere, which revealed worm burden reductions of 84% and 27.5% at 300 mg/kg and 150 mg/kg treatment doses in rats, respectively [29]. In our study, we focused on evaluating the combinatorial treatment efficacy of CUR+PZQ. To better understand the synergistic effect of CUR+PZQ, a slightly lower dose (250 mg/kg), which is anticipated to have a lesser anthelmintic effect than those demonstrated by 300 mg/kg, was selected. Overall, CUR+PZQ improved parasite clearance and promoted the resolution of hepatobiliary tissue damage.

To date, several combinatorial studies involving PZQ have been reported with interesting findings, whether they be synergistic or antagonistic. Combining PZQ with OZ78 and artemether enhanced parasite burden compared to PZQ monotherapy alone, while combinatorial therapy using PZQ and artesunate acted synergistically against *C. sinensis* infection [30]. Additionally, combining artesunate and tribendimidine also led to the rapid expulsion of *C. sinensis* worms from infected rats at 3 dpi [31]. However, these findings conflict with our earlier study, which demonstrated the lack of trematicidal effect of PZQ against CsMC and CsNEJs in hamsters [14]. Such differences are thought to arise from animal sensitivity to parasites. In support of this notion, Sohn et al. [32] have reported differing resistances to *C. sinensis* reinfection in various experimental animals, including rodents, pigs, and others. Despite the conflicting reports of synergistic and antagonistic effects demonstrated by combinatorial PZQ treatment, we anticipated that CUR+PZQ would induce a synergistic effect and resolve *C. sinensis* infection. A previous study revealed that CUR possesses molluscicidal and cercaricidal activities [33]. This highlighted the CUR potential as a supplementary drug for PZQ, thus ensuring the trematicidal effect against both larval and adult stages of *C. sinensis*. Furthermore, a large number of studies have documented the enhanced antiparasitic effect of drugs when combined with CUR against a wide array of parasites, including *Plasmodium falciparum* [34,35], *Leishmania donovani* [36], *Cryptosporidium parvum* [37], *Fasciola gigantica* [38], and others. Based on these earlier findings, we anticipated that the trematicidal effect of PZQ would be significantly enhanced by CUR co-formulation, and this therapeutic regimen would induce the killing of both larval and adult *C. sinensis* parasites.

There are two limitations to the present study. First, while CUR can be used to enhance drug efficacy, it is worth mentioning that prolonged CUR usage can lead to acute liver injury [39]. Because this study did not investigate the effect of repeated CUR or CUR+PZQ administration for clonorchiasis treatment, the possibility of CUR-induced hepatic damage cannot be ruled out. Furthermore, one of the major issues hindering the clinical development of CUR is its poor bioavailability and pharmacodynamic properties [40,41]. In humans, individuals taking high doses of CUR were reported to possess plasma CUR concentrations in the nanomolar range [42], far below the concentrations tested in the present study. The pharmacokinetic differences between CUR and PZQ could also lead to inconsistent drug uptake, implying suboptimal amelioration of hepatobiliary pathologies caused by *C. sinensis*. As such, attempts to address these limitations should be prioritized.

In conclusion, while CUR possesses limited efficacy against *C. sinensis*, it synergized well with the anthelmintic drug PZQ. Combining CUR with PZQ not only improved parasite burden reduction but also contributed to lessening parasite-induced hepatobiliary pathologies, especially during the early stage of infection.

## Data Availability

The original contributions presented in the study are included in the article/Supplementary Material, further inquiries can be directed to the corresponding author.

## Supplementary Materials

The following supporting information can be downloaded at: https://www.mdpi.com/article/10.3390/pharmaceutics16121550/s1, Figure S1. The correlation between XTT absorbance and parasite numbers.
