# Supplementary material for: Combinatorial Treatment with Praziquantel and Curcumin Reduces Clonorchis sinensis Parasite Burden and Clonorchiasis-Associated Pathologies in Rats
Source: Pharmaceutics. 2024 Dec 3;16(12):1550. doi: 10.3390/pharmaceutics16121550 (PMC11678916; doi:10.3390/pharmaceutics16121550)
Supplement: Supplementary file 1 [file pharmaceutics-16-01550-s001.zip › pharmaceutics-3256007-supplementary.pdf]

Supplementary Material

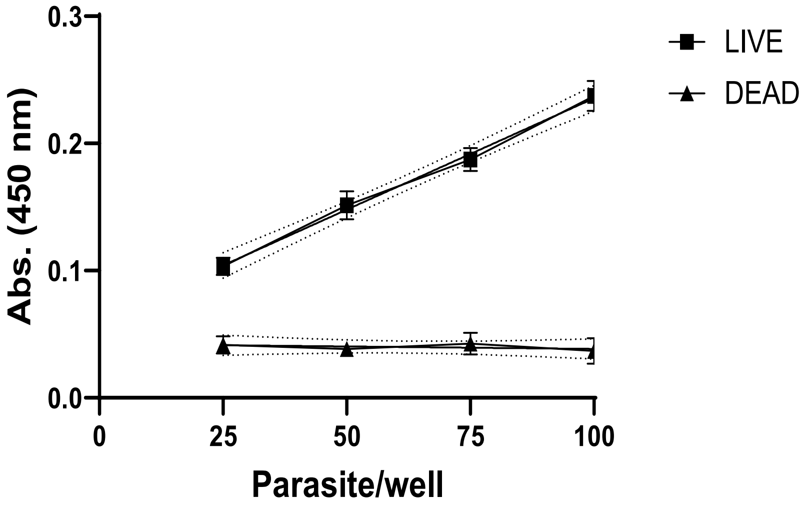

Figure S1. The correlation between XTT absorbance and parasite numbers. The correlation was determined by inoculating varying numbers of living or heat-killed *C. sinensis* adults into 96-well plates. A positive correlation between parasite numbers and optical density readings was found, as indicated by the linear regression line.
